# A hybrid pipeline for carotid artery segmentation using YOLOv11n and contour models

**DOI:** 10.1038/s41598-026-41007-2

**Published:** 2026-03-23

**Authors:** Gerges M. Salama, Mohammed Safy, Dina A. Hassanin, Ashraf A. M. Khalaf, Mahmoud Khaled Abd-Ellah

**Affiliations:** 1 Department of Electrical Engineering, Faculty of Engineering, Minia University, Minia, 61111 Egypt; 2 College of Computing and Information Technology, Arab Academy for Science, Technology and Maritime Transport (AASTMT), Smart Village, Giza, 2401 Egypt; 3 Electrical and Computer Engineering Department, Minia Higher Institute of Engineering and Technology, New El-Minya, Minia Egypt; 4 Faculty of Artificial Intelligence, Egyptian Russian University, Cairo, 11829 Egypt

**Keywords:** Carotid artery, Ultrasound imaging, Segmentation, Deep learning, Active contour, Stroke, Computational biology and bioinformatics, Diseases, Engineering, Neurology, Neuroscience

## Abstract

Carotid artery segmentation is critical for determining the degree of vascular disease, and for recommending treatment options. Early detection of carotid atherosclerosis is critical for preventing stroke. Stroke-related brain damage can cause deficits in speech or vision, and large strokes can be fatal. However, automatic segmentation of the carotid artery lumen remains difficult due to the low quality of US images, and the existence of stenosis, jugular veins, and abnormalities in carotid images. This article presents a hybrid pipeline for segmenting both carotid transverse and longitudinal lumens without any user interaction. This hybrid pipeline starts with automatically localizing the carotid artery lumen in the transverse and longitudinal sections via YOLOv11n. Then, a multistage preprocessing framework was applied to the transverse section before its lumen was segmented by the active contour. For the longitudinal section, an automated padded mask was generated to guarantee reliable initialization for Chan-Vese level-set evolution. A paired t test validated the relevance of the proposed modules (*p* < 0.0001). The proposed multiphase segmentation pipeline achieved a Dice index and accuracy of 94.9% and 97.7%, respectively, for the longitudinal section and 90.8% and 99.6%, respectively, for the transverse section. A comprehensive ablation analysis has shown that numerical stability depends on the YOLOv11n localization phase. The system attained near real-time inference for the carotid transverse section (< 1 s) despite being evaluated on low-end hardware, demonstrating its computational efficiency and promise for clinical integration.

## Introduction

The primary blood vessels in the neck that carry blood to the brain, neck, and face are known as carotid arteries^1^. The carotid artery narrows and thickens as a result of elevated cholesterol and the deposition of lipid molecules on blood vessel walls, inducing arterial constriction and obstructing blood flow to the brain. The constriction or occlusion of the arteries in the neck region can hinder or interrupt the delivery of blood to the brain, resulting in strokes^2^. Manifestations of constriction of the arteries in the neck do not show at the start of sickness and occur only after a stroke. As a result, beginning at the age of 50, frequent visits to a vascular expert are required to discover issues early, especially for diabetic patients, smokers, and heart sufferers. Heart disease is the primary cause of death worldwide, followed by stroke. Someone dies of a stroke every three minutes and 30 s on average according to the American Heart Association^3^.

Carotid artery disease is one of the most common disorders worldwide. Because of insufficient knowledge and prompt diagnosis of those at risk of developing atherosclerosis, there has been an increase in cases detected and reported in advanced stages of carotid artery disease after the emergence of major health issues^4^. As a result, screening programs are needed to detect the early stages of carotid artery constriction, which helps to control the progression of atherosclerosis and avoid suffering from stroke or heart attack^5^. Vascular segmentation techniques have evolved in various medical imaging modalities to diagnose cerebrovascular disorders. MRI/MRA is a valuable tool for assessing intracerebral carotid arteries and cerebral circulation in noninvasive, radiation-free, and iodized examinations^6^. Various studies have leveraged the U-Net family and multilevel residual dual-attention networks to accomplish accurate volumetric segmentation of cerebral arteries^7–9^. Additionally, incorporating multiscale dual-attention mechanisms and context-aware preprocessing improved the quantification of intracranial aneurysms before and after therapy^10–12^. Although carotid medical imaging takes several forms, carotid ultrasound (US) imaging is a painless, noninvasive procedure that uses sound waves to examine carotid arteries for blood flow^13^. Carotid US images are used to examine the constriction or occlusion of arteries if a patient has chronic problems or perhaps some cases of stroke^14^.

There are numerous difficulties associated with meticulous and precise manual/automatic segmentation of the US carotid lumen. Manual segmentation is labor intensive, and competence is needed to locate and delineate the carotid lumen. Identifying atherosclerotic plaques and lesions in complicated carotid artery geometries can be problematic. There is a paucity of experienced radiologists evaluating carotid vessels. Additionally, carotid US images include speckle noise and inadequate contrast. The segmentation process is difficult because of the varying echo qualities of the carotid artery for different US imaging devices. Although end-to-end deep learning models have demonstrated potential, noise-induced instability and a lack of transparency frequently limit their use to carotid US images. Therefore, an automated strategy is needed to segment the carotid lumen accurately and robustly in US images, which is an essential starting point for measuring the lumen region, atherosclerotic area, and other relevant parameters. This paper presents a fully automated system for segmenting both carotid artery sections depending on the robust localization phase, noise suppression framework, and automated mask generation. All the applied phases were validated through rigorous ablation and statistical testing to ensure clinical dependability. The proposed hybrid pipeline was chosen to combine the lightweight YOLOv11n localization model, which provides an automatic initial contour, with a lightweight geometry-based refinement active contour. This combination provides a fully automated system, simple architecture, and accurate localization and segmentation. The main contributions of this article can be summarized as follows:

1. A synergistic hybrid pipeline is presented that consists of a deep learning YOLOv11n model to localize the carotid artery lumen, combined with processing and initialization techniques for both carotid sections separately before the final segmentation approach is applied. This hybrid pipeline leverages the strengths of both models to achieve efficient segmentation of the carotid artery lumen.
2. Our hybrid pipeline completely automates the challenging initialization process for carotid artery transverse and longitudinal lumen segmentation, constructing a highly refined initial mask with no user involvement.
3. A specialized improvement pipeline built specifically to overcome the constraints of carotid transverse US images. The sequence stages, including bilateral filtering, CLAHE, gamma correction, NLM denoising, and high-boost sharpening, minimize speckle noise, increase local contrast, and clarify sharp edges, resulting in an optimal energy environment for the active contour model.
4. Automatic generation of a second mask for segmenting the carotid longitudinal lumen, which in turn contributed to the accuracy and robustness of the carotid lumen Chan-Vese segmentation and the failure to segment other parts of the image with the same opacity density, such as the jugular vein, as was the case in other studies.
5. Our suggested end‒to‒end hybrid pipeline significantly improves the lumen segmentation accuracy of transverse and longitudinal carotid sections, which are often challenging to achieve because of inadequate contrast, excessive speckle noise, and the existence of other veins or objects in carotid images.

## Related work

### Carotid transverse section segmentation

Many studies have investigated the segmentation of the carotid artery lumen in transverse sections, but in most of these studies, the initial seed for the segmentation process was determined manually. A deformable contour technique with just one seed point as manual initialization was presented by Mao et al.^15^ to segment the carotid B-mode US transverse lumen. Deformable contour and morphological operations are initiated via entropy mapping, which is based on local statistical data such as the mean gray level and standard deviation of the gray level. van’t Klooster et al.^16^ introduced a semiautomated model to segment the inner and outer walls of carotid 3D MRA images via user interaction. They initialize a tube structure involving the formation of several rings with control points oriented perpendicular to the identified carotid vessel axis. Yang et al.^17^ proposed a semiautomated technique for segmenting the inner and outside CCA walls via morphology combined with GVF snake algorithms. Their segmentation technique was applied to 110 transverse CCA images and achieved Dice coefficients of 90.3% and 92.7% for adventitia and lumen segmentation, respectively. They depend on the previous lumen ellipse to manually extract the seed points used for the CCA adventitia segmentation.

A semiautomated procedure for CCA lumen segmentation that utilizes level sets guided by regional intensity and spatial information was employed by HuiTang et al.^18^. The initial points were set manually near the CCA lumen to achieve the lumen segmentation procedure. For the CLS2009 challenge, the authors used 56 CCA images from a freely accessible source to achieve a 90.2% Dice index. Ukwatta et al.^19^ faced difficulty in segmenting low-contrast 3D US carotid transverse images via sparse field level sets. Therefore, they were unable to segment the artery automatically, so four anchor points close to the artery edges were manually used to facilitate the segmentation process. Gaussian filtering and speckle-removing anisotropic diffusion filtering are used to mitigate noise in 3DUS images after the intensity of the whole image has been normalized to increase the clarity of the internal and exterior carotid artery borders. They applied their procedure to 21 3D US carotid transverse images, achieving a Dice similarity rate of 90.6 ± 5% for the carotid lumen. Andres et al.^20^ presented a semiautomatic procedure that uses surface graph cuts to segment normal and pathological carotid transverse images. Their procedure cannot perform carotid lumen segmentation without user interaction to establish the initial setup points. Twelve carotid arteries from six healthy subjects and patient participants whose carotid images were acquired were used to test the technique. They achieved Dice indices of 84% and 66.7% for normal and atherosclerotic volunteers, respectively.

Md. Murad Hossain et al.^21^ proposed a semiautomated algorithm using a distance regularized level set to segment the lumen of 3D US carotid transverse images. A selected group of transverse slices with an intersection distance of 4 mm are used for manually arranging points on their boundaries to initialize the procedure. They achieved a Dice index of 89.82% for 5 patients with atherosclerosis rates greater than 50%. They also applied the same segmentation technique with the addition of ellipse fitting contours and stop-based energy criteria. They used 10 subjects with 3D US carotid transverse images, and 6 points were selected by the user as the segmentation initialization step, achieving a Dice index of 88.78% for the carotid lumen^22^. CCA detection and segmentation were applied using an echogenicity-based algorithm, and the US inferior formation was performed by Narayan et al.^23^.The region of interest was identified that characterizes the CCA using hypoechoic anatomical images and then segmented via least square ellipse fitting.The Otorhinolaryngology Hospital provided them with a dataset of 41 images and a manual segmentation of each image, obtaining a Dice similarity of 87.4%. Arias-Lorza et al.^24^ introduced a semiautomated approach to segment 3D MRAs of 57 carotid artery images via coupled surface graph cuts, achieving a Dice overlap of 89% for the carotid lumen.

Hemmati et al.^25^ proposed a semiautomated segmentation approach using 3D Hessian and level set methods to segment carotid data from 14 CTA volumes. The carotid gray level homogeneity was improved via mean shift smoothing, and the artery centerlines were applied with the aid of three manually applied initial points, achieving a Dice value of 85%. Jodas et al.^26^ developed an automated approach for locating the lumen in carotid artery MR images. The K-means approach was assessed via a circularity index, with the one with the highest value signifying the possible lumen region. The boundaries of the identified lumen region were then fine-tuned via an active contour technique. Their recommended approach achieved a Dice coefficient of 0.78 ± 0.14 in 181 postcontrast 3DT1-weighted and 181 proton density-weighted MR images. Luo et al.^27^ suggested an approach for semiautomatically segmenting the carotid artery lumen on weak boundary time-of-flight (TOF)-MRA images. Their suggested technique incorporated a dual adaptive threshold into the classic level set method (DATLS). Their suggested approach was evaluated via TOF-MRA images with manual outlining as the ground truth, yielding a Dice coefficient of 0.88 ± 0.07 and a reduced mean contour distance (MCD) of 0.48 ± 0.37 mm. Their dependency on image quality, manual start-up, and testing restricted to TOF-MRA scans are its primary drawbacks. Zhu et al.^28^ introduced a carotid transverse section segmentation algorithm using enhanced surface graph cuts that used reduced flow line sampling, and a semiautomatic initial contour was used. Their dataset was collected from MR images of individuals with atherosclerotic carotid arteries and achieved a Dice coefficient of 89.6% for lumen segmentation.

Recent studies have shown that the use of deep learning in carotid transverse section segmentation has made significant progress. However, the results of deep learning depend heavily on image conditions, including image quality and quantity, and on any pre- or postprocessing applied to increase the efficiency of these models. Liu^29^ proposed a deep learning model called CANet for segmenting the carotid lumen intima and media adventitia boundaries and then measuring carotid stenosis severity. ICA-SAMv7 was used to segment the carotid artery by combining YOLOv7-based localization with a fine-tuned segment annealing model (SAM)^30^. The SAM performs insufficiently for segmenting the carotid artery and needs supplementary localization and parameter-efficient fine-tuning to obtain satisfactory results. Zhou et al.^31^proposed a semiautomated deep learning procedure to segment 144 3D US carotid transverse images. U-Net was applied to segment the carotid lumen, enabling end-to-end training of the model for pixelwise classification and achieving a Dice index of 92.8%. A manually cropped rectangular region of 224 3D US internal carotid artery (ICA) images was used to segment the carotid lumen via two-channel U-Net and adaptive triple Dice loss. The Lumen intima boundary of the ICA had a Dice coefficient of 89% ± 8.1%^32^. Xie et al.^33^ developed a deep convolutional U-Net framework for internal carotid artery lumen segmentation. They utilized 302 images from 98 cases, trained them through 10-fold cross-validation, and obtained approximately 95% segmentation accuracy via the basic U-Net architecture. CSWin transformer models are used to segment the carotid media-adventitia and lumen-intima in 3D US images^34^. Their application to standard 2D clinical US applications is limited by their dependence on volumetric imaging, specialist slice-wise annotation, and computationally difficult architectures. Jonnala et al.^35^ introduced a deep learning algorithms to assess transverse lumen segmentation by comparing U-Net, SegNet, and an enhanced U-Net architecture. Their algorithm was applied to 2165 transverse images, achieving a Dice coefficient of 87.03%. An unsupervised shape and texture-based generative adversarial tuning architecture was presented by Chen et al.^38^ to segment the LIB of 3D US carotid images. achieving a Dice coefficient of 85.7%.

### Carotid longitudinal section segmentation

Fewer studies have addressed carotid artery longitudinal section segmentation than transverse segmentation because of the scarcity of longitudinal datasets and their limited number of subjects^36^. The Hough transform was applied by Golemati et al.^36^ to segment the carotid B-mode US longitudinal lumen, with the resulting lumen assumed to have two straight parallel walls. Ten healthy patients and four atherosclerosis-affected subjects were included in their dataset. They obtained a specificity and accuracy greater than 96%. Their approach assumes that the longitudinal lumen is two straight lines, which is not suitable for all longitudinal section imaging. Md. Murad Hossain et al.^37^introduced a semiautomatic segmentation approach for the carotid artery longitudinal lumen. Their methodology involves manually selecting the border points on the longitudinal slice where the bifurcation topology is most easily visible, in conjunction with a distance regularization level set evolution. Five patients with carotid atherosclerosis participated in the evaluation, and the findings showed a Dice similarity coefficient of 86.4%. C. P. Loizou et al. introduced a semiautomated integrated system to delineate the common carotid artery via snake segmentation. Snake segmentation adjusts to the lumen boundary once the technician supplies initialization. Three separate databases, including 300 longitudinal 2D US images of the CCA bifurcation, were used for validation. Kumar et al. ^38^ segmented the carotid lumen in longitudinal sections via active oblongs. They used a combination of binary thresholding and the Hough transform to obtain the initial rectangle inside the artery. Their initialization method is not effective, as they mentioned where it drops with weak boundary contrast carotid images. They used the SP lab dataset of 84 longitudinal carotid sections and achieved a Dice index of 93.35%. Gagan et al.^39^ introduced a basis spline-based active contour approach to segment the carotid longitudinal lumen. The gradient descent strategy was applied to minimize the local energy function to segment the carotid lumen, and Green’s theorem was also applied for additional optimization. Their technique was applied to 84 images of SP Lab longitudinal sections, with a Dice index of 91.7%.

The application of deep learning techniques in longitudinal section images of the carotid artery is relatively limited compared with that in transverse sections because of the scarcity of datasets and the small number of images they contain. Furthermore, deep learning applications have focused more on segmenting carotid artery plaques than on segmenting the carotid lumen itself. A modified U-Net structure was developed for automatic plaque segmentation in carotid longitudinal sections, allowing plaque region computation^40^. Their model was developed and verified on 144 patients, with Monte Carlo cross-validation and observation-based annotations conducted manually as ground truths. Kiernan et al. ^41^ proposed a segmentation algorithm for the carotid artery longitudinal lumen via Mask R-CNN. The network was trained via annotated carotid ultrasound images of 101 patients in which specialists manually defined the ground truth lumen borders. The technique produced pixel-level lumen masks after initially using bounding box prediction, obtaining intersections over unions of 0.81 and 0.75 for bounding box detection and lumen segmentation, respectively. A TransUNet–Vision transformer was applied for plaque segmentation and echogenicity characterization in longitudinal US images^42^. However, their segmentation results were limited and heavily dependent on plaque echogenicity, reflecting ongoing challenges in defining plaque borders in noisy B-mode images, despite high classification accuracy. A deep learning approach for longitudinal carotid lumen segmentation was proposed by Biswas et al.^43^. Their system used triple ground-truth annotations from 407 ultrasound images to train an FCN decoder for lumen reconstruction and a 13-layer CNN encoder for extracting features.

## Methodology

**Fig. 1:**
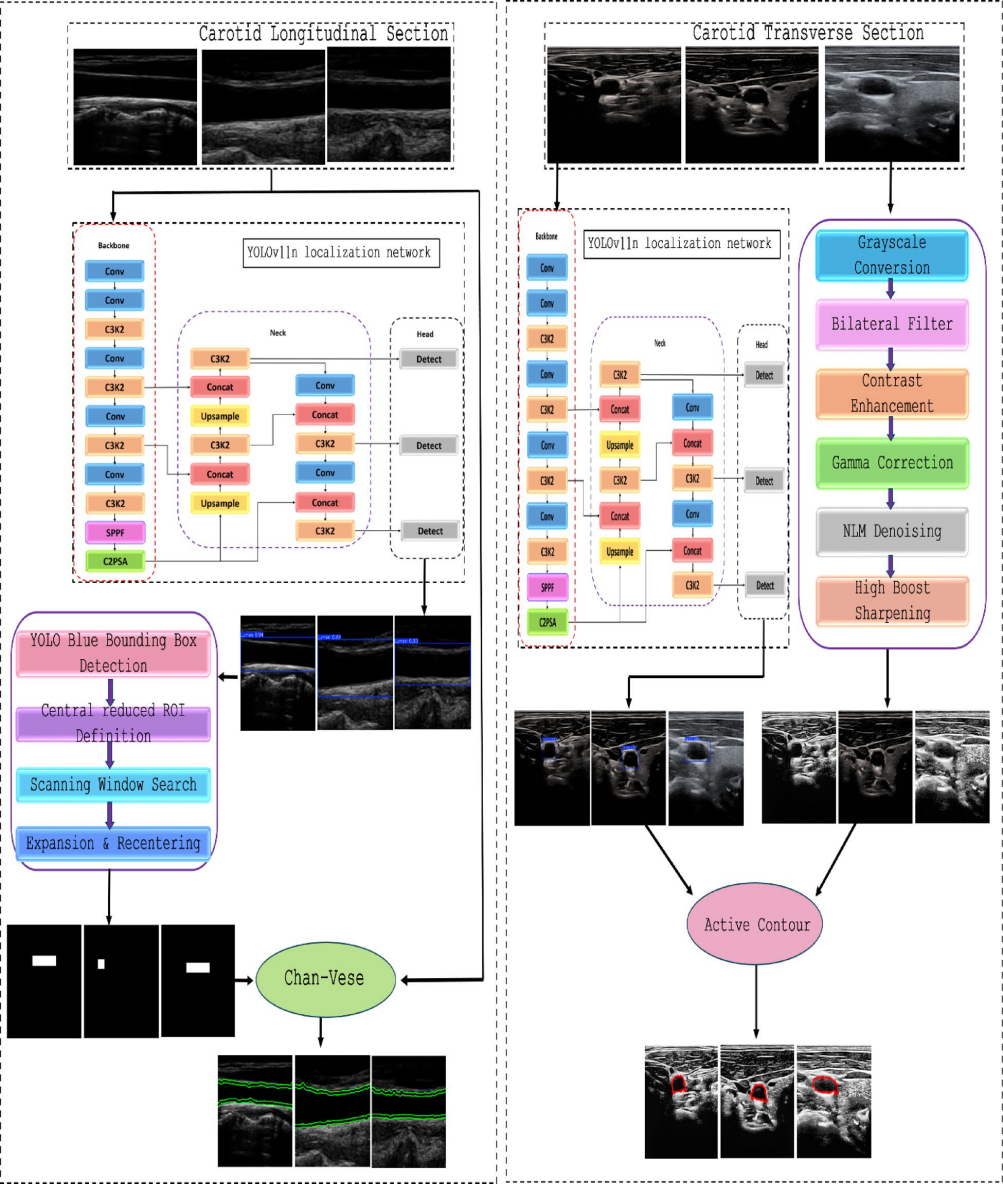
A flowchart of the proposed hybrid pipeline for carotid artery lumen segmentation. The carotid lumen segmentation for both transverse and longitudinal sections has three different phases: the localization phase, an intermediate phase, and the final contouring phase.

The hybrid pipeline methodology consists of three main phases for each carotid section, as shown in Fig. 1. The carotid longitudinal and transverse sections have the same first localization phase, which automatically constructs the initial bounding box for both carotid section lumens. For the transverse section, essential enhancement stages are applied before the active contour segmentation approach. For the longitudinal section, a second bounding box is generated depending on the first bounding box to accurately segment the longitudinal lumen with the Chan-Vese model.

### Carotid localization and feature map interpretation

**Fig. 2:**
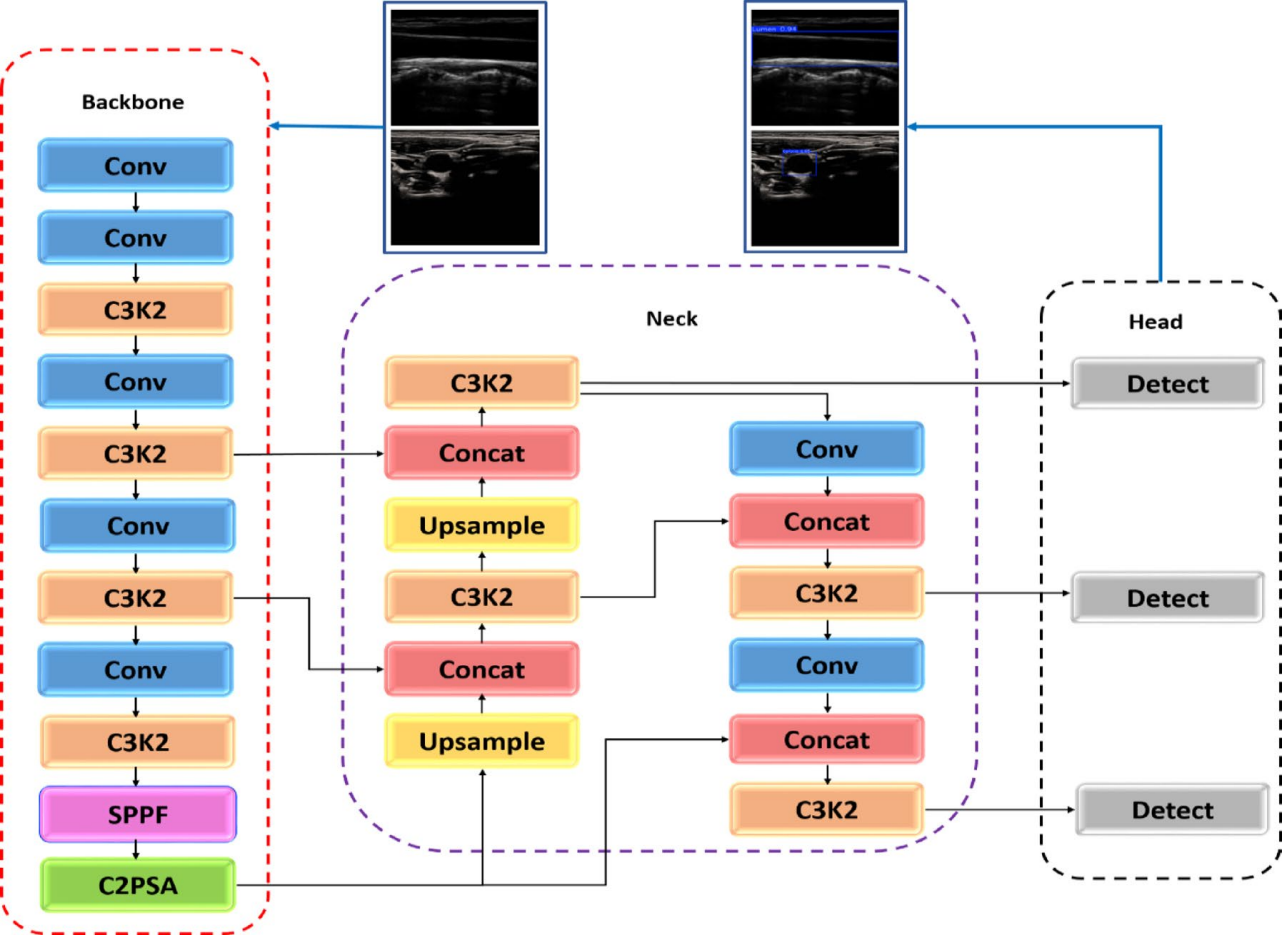
Structural diagram of the YOLOv11n localization network.

Carotid lumen localization for both transverse and longitudinal sections was performed via YOLOv11n. YOLO) You Only Look Once) models have made a difference in the field of object detection, and YOLOv11 has outperformed and made significant progress compared with all previous versions and the version that came after it, which is YOLOv12 ^44^. YOLOv11 has undergone numerous developments and updates its structure and training, enabling it to instantly detect objects with great accuracy, speed, and efficiency. Two datasets containing a different section of the carotid artery represented in the longitudinal section and the transverse section of the artery were used separately to train and improve the performance of YOLOv11n (Nano version). YOLOv11n was fine-tuned on both carotid datasets, and the previously trained model was optimized on the carotid artery datasets. This allowed for accurate detection of the boundary box of the artery lumen, facilitating and improving the subsequent segmentation phase of the carotid lumen. The YOLOv11n architecture consists of three main stages, as shown in Fig. 2: the backbone (marked by a red dashed box), whose main function is to extract the distinctive features of the carotid lumen through convolutional layers. The second stage, the neck (marked by a purple dashed box), is an intermediate stage between the backbone and the final stage, which collects and enhances features at multiple scales to determine the different sizes of the carotid artery. Finally, the head stage (marked by a black dashed box) performs the prediction process and generates the final outputs of the detection process, determining the boundary boxes defining the carotid lumen and confidence levels.

The backbone includes a series of convolutional layers that reduce input image samples and C3K2 blocks, which are key improvements that set this model apart from its predecessors. The C3K2 blocks feature two small convolutions.

instead of one large convolution, which significantly speeds up feature acquisition and increases computational efficiency. YOLOv11 also includes the introduction of a cross-stage partial with spatial attention (C2PSA) block, which also improves the precision of focusing on important regions in images. This has contributed to the detection of small objects of different sizes^45^, while the spatial pyramid pooling-fast (SPPF) block has been retained. The data pass through the layers that make up the backbone, which helps systematically lessen its dimensions and obtain the.

basic features that will be collected later in the neck. The neck’s combination and integration of these features is performed through layers of concatenation (Concat), upsampling accompanied by C3k2 blocks, ensuring efficient integration to accurately detect carotid artery lumens of varying sizes. The head’s mission is to generate the final predictions for YOLOv11n by generating bounding boxes and confidence ratings. The detection layers are arranged on three scales: tiny, medium, and large. They process all different levels of feature maps, ensuring that different carotid artery locations and sizes are detected with high accuracy and efficiency.

In this work, YOLOv11n was fine-tuned to accurately detect the carotid lumen in longitudinal and transverse sections, generating a bounding box containing the entire carotid lumen. Modifications to this model, combining the aforementioned components of image analysis and feature extraction with transfer learning, enabled us to generate an accurate initial automated contour, which then assisted in segmenting the artery via an active contour or Chan-Vese. Several factors contributed to the accuracy and immediate performance of this model, including the previously mentioned components of image analysis, feature extraction, the application of transfer learning to transverse and longitudinal datasets and a precise ground truth that enabled the model to accurately learn the artery location, as well as robust training and validation. This integration helped in generating an accurate and automated initial contour, which in turn aided in segmenting the carotid lumen using the active contour in the transverse section or Chan-Vese in the longitudinal section.

**Fig. 3:**
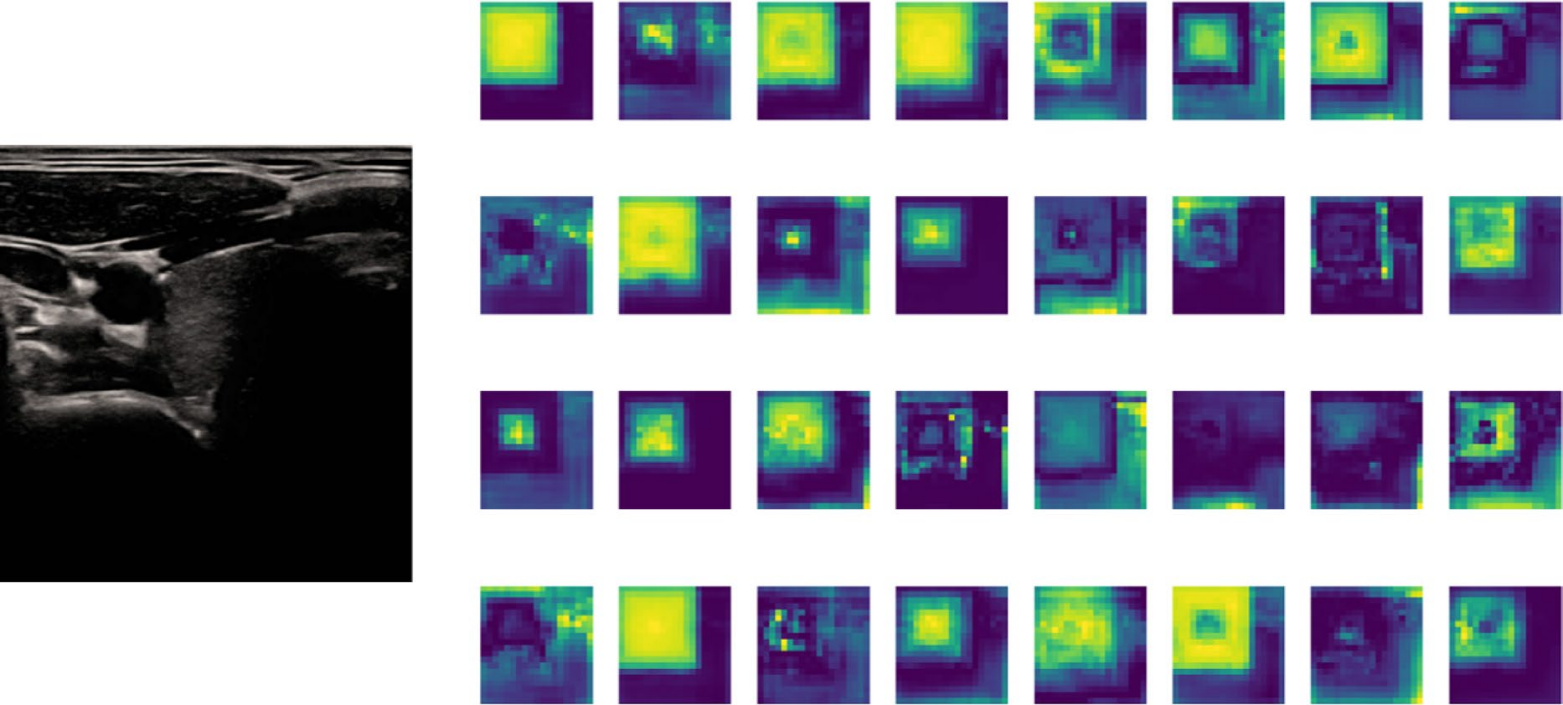
Layer-wise feature map visualization for transverse carotid lumen localization.

**Fig. 4:**
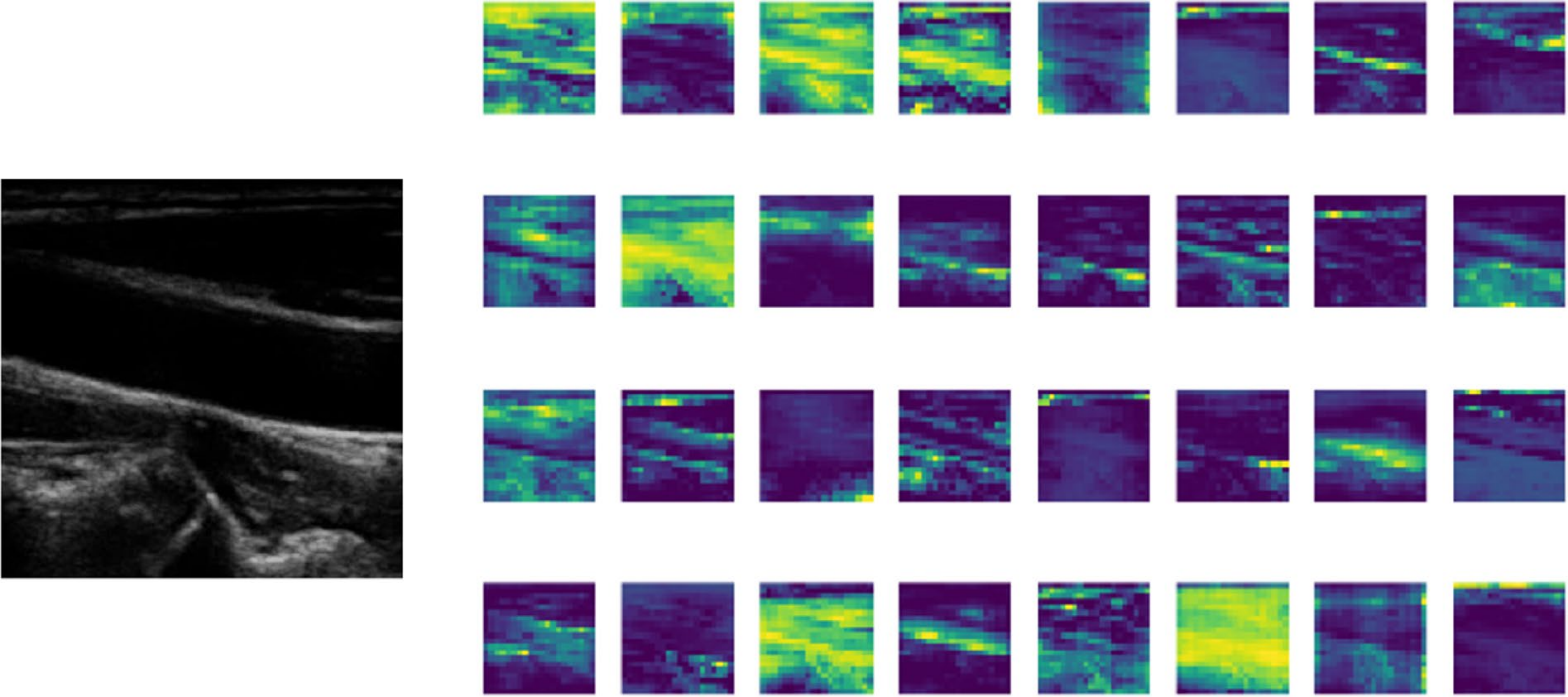
Layer-wise feature map visualization for longitudinal carotid lumen localization.

Layer-wise feature map analysis was performed to improve the interpretability of the model. The model effectively reduces nonvascular textural noise and concentrates high-intensity activations on the arterial borders by viewing the internal activations of the YOLOv11n backbone, especially inside the C3k2 and C2PSA modules. Layer-wise depiction of the feature maps was performed, as shown in Figs. 3 and 4, to better comprehend the model’s internal decision-making process. The borders of the carotid lumen exhibit highly localized spatial activations in the final representation, which form a tubular alignment in the longitudinal plane and a circular pattern in the transverse plane.

### Carotid transverse section segmentation

Before the active contour is employed to segment the carotid transverse section, a preprocessing framework is applied. This extensive preprocessed framework is an essential component of the segmentation process, not only a preamble. The active contour technique involves a spline that bends to meet the object borders while minimizing energy. It is strongly reliant on the image’s energy landscape, which is made up of features such as intensity gradients. This landscape is gradually improved via the proposed enhancement stages.

### Carotid transverse section preprocessing

We implement a series of preprocessing steps on the original common carotid US images of 709 × 749 × 3 dimensions, which are converted to grayscale, to reduce complexity and improve the efficiency of the subsequent steps. These sequential preprocessing steps contributed significantly to the efficiency of the next stage, which is segmentation via an active contour. The importance of image preprocessing was represented in addressing problems presented in common carotid US images, such as speckle noise, low contrast, and unclear carotid lumen boundaries. These subsequent preprocessing steps prepare the image and make it ideal for the next segmentation stage, as discussed in the next subsections.

### Bilateral filter

A bilateral filter was applied to the carotid artery grayscale images to reduce speckle noise and smooth the carotid images as much as possible while preserving the outer boundary edges of the carotid lumen. This nonlinear filter estimates each pixel’s intensity by taking the weighted average of the intensity values of the pixels surrounding it^46^. Both the radiometric variation and the geometric distance influence the weights. The bilateral filter results an output I(x) as shown in Eq. (1) for a pixel at location x, with normalization factor W_x_, kernel of spatial Gaussian $\:{G}_{{\sigma\:}_{s}}$, Gaussian kernel range $\:{G}_{{\sigma\:}_{r}}$, and spatial neighborhood of pixel x is S .

1 $$\:I\left(x\right)=\frac{1}{{W}_{x}}\sum\:_{z\:ϵS\:}{G}_{{\sigma\:}_{s}}(\:\:⃦x-z\:\:⃦){G}_{{\sigma\:}_{r}}\left(\:\right|\:I\left(x\right)-I\left(z\right)\left|\:\right)I\left(z\right)$$

### Contrast enhancement

The Bilateral filtered carotid images local contrast is enhanced using Contrast Limited Adaptive Histogram Equalization (CLAHE)^47^. CLAHE of 8 × 8 tiles divide the carotid transverse image to little areas called tiles and then increase the contrast of each tile separately, this makes the output region histogram roughly resemble a predetermined histogram. The Clip Limit was employed at 0.02 to avoid over amplification of noise, which might happen in areas that are almost homogeneous. The CLAHE enhanced the local contrast of the carotid outer lumen boundary and make it distinct which helps in avoiding impeding of the segmentation phase.

### Gamma correction

Gamma correction is employed after contrast enhancement stage using CLAHE to modify the entire carotid transverse images luminosity and intensity. This non-linear Gamma approach^48^ adjusts the carotid images luminous levels to account for changes in brightness or display. The transformation function that was applied as shown in Eq. (2) with gamma value ɣ, constant k, and the entered CLAHE pixel value from [0, 1] normalized as I_clahe_. A Gamma of 1.2 was chosen which helps carotid mid bright areas to gently brightened without overpowering the brightest areas achieving a more consistent pattern of intensity for active contour segmentation.

2 $$\:{I}_{gamma}=k\:.\:{I}_{clahe}^{ɣ}$$

### Non-local means denoising

After applying Gamma correction, a more potent denoising phase that is provided by non-local means denoising (NLM)^49^ successfully smooth the textures inside the carotid lumen boundary area by effectively eliminating granular noise, preventing the active contour from becoming stuck on small tissue flaws. NLM takes into account a considerably larger searching window and calculates a weighted average of all pixels in this window (our search window size = 21, and a comparison window or patch = 7), in contrast to local filters which average pixels in a narrow neighborhood. As shown in Eq. (3), the NLM denoised output image I_nlm_, I_q_ is the input image at pixel q from Gamma phase, normalization factor N_p_, with patches P_p_ and P_q_, $\:{\:\:\:⃦{P}_{p}-{P}_{q}\:\:⃦}_{2}^{2}$ is the Squared Euclidean distance between patches, and h is the filtering parameter which controls the smoothing amount.

3 $$\:{I}_{nlm}=\frac{1}{{N}_{p}}\sum\:_{q}{e}^{\frac{-{\:\:\:⃦{P}_{p}-{P}_{q}\:\:⃦}_{2}^{2}}{{h}^{2}}\:}{I}_{q}$$

### High-boost filtering

The Final chosen stage in the pre-processing framework is High-Boost Filtering^50^ to make the carotid lumen boundary more strong and sharpen that serves as a distinct path for the active contour segmentation algorithm. The high-frequency portions that correlate to carotid lumen outer edges and small features becomes more apparent using High-Boost approach. It functions to obtain I_sharpen_ by taking a blurry image I_blur_ and removing it from the NLM output image I_nlm_, then adding the outcome back to the input NLM image as shown in Eq. (4). A Gaussian filter is used to create the blurred image, and α is the scaling factor that regulates the sharpening level. The carotid lumen edge input data was.

amplified using an α of 1.5, resulting in distinct, and robust lumen boundaries that are optimal for the active contour algorithm to follow.

4 $$\:{I}_{sharpen}=\:\:{I}_{nlm}+\:\alpha\:\:.\:({I}_{nlm}-{I}_{blur})$$

### Carotid transverse lumen contouring

The active contour model^51^ is used to segment the carotid transverse section boundaries. Active contour approach which acts as energy minimizing spirals is initiated on US carotid enhanced images and undergo repetitive deformation in order to minimize a specified energy function and latch onto the transverse carotid lumen boundaries. The segmentation process of the transverse section begins by determining the initial mask, on which the efficiency of the contour depends. This initial mask was identified by determining the color channel intensity, with red and green channels being less than 100 and blue channel being larger than 150 of the YOLOv11n Localized bounding box of the transverse section. Then, morphological operations are carried out to ensure a solid box, such as bwareafilt, which keeps the most noticeable linked region, and imfill, which fills in any small gaps found in the blue box area. The bounding box coordinates were then retrieved using regionprops as [w, h,x, y], which stood for [width, height, top corner, left corner]. The active contour approach repeatedly deforms a contour parameterized curve C(s) to reduce the global energy functional for enhancing the initial bounding box. This assists the contour to accurately smash to the high-gradient boundaries in the enhanced transverse image. The preceding integral defines the full energy functional Esnake that this approach reduces where α, β, Ɣ are weighting coefficients that regulates the contour’s tension and rigidity. The contour 1st and 2nd derivatives are $\:{C}_{s}\left(s\right)$ and $\:{C}_{ss}\left(s\right)$ and $\:I\:\left(C\left(s\right)\right)$ represents each curve points image intensity.

5 $$\:{E}_{snake}\:=\:{\int\:}_{0}^{1}\left(\:\frac{1}{2}\right(\alpha\:|{{C}_{s}\left(s\right)|}^{2}+\:\beta\:\left|{{C}_{ss}\left(s\right)|}^{2}\right)-Ɣ\:|\nabla\:\:I\:({C\left(s\right)\left)\right|}^{2})ds$$

### Carotid longitudinal section segmentation

Although YOLOv11n is an accurate and effective in the common carotid artery longitudinal lumen localization with the blue bounding box shown, this blue box contains not only the carotid lumen to be segmented but also surrounding tissue, noise, and other components with the same intensity as the lumen. Some images also contained the jugular vein in the blue box along with the carotid artery lumen. Both had the same opaque intensity due to the presence of blood, which prevents them from being reflected by ultrasound waves. Preparing the appropriate bounding box is a critical step for the accuracy and robustness of the subsequent segmentation technique using Chan-Vese.

### Automatic generation of segmentation based longitudinal mask

**Fig. 5:**
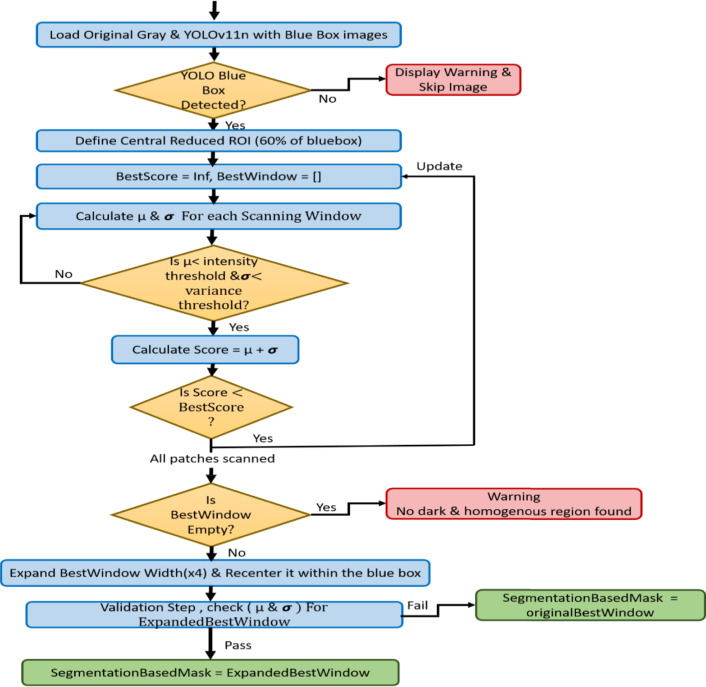
Generation of the Chan-Vese Segmentation based Longitudinal mask.

It was necessary to generate a second box based on the YOLOv11n box to ensure the Chan-Vese energy function was minimized to accurately and efficiently segment the correct carotid artery lumen. The segmentation based bounding box was generated using the following technique steps as shown if Fig. 5. First, the yolo blue bounding box was detected by identifying its color channel intensity considering as the blue channel to be greater than 150 and red and green channels are less than 100. Then, to guarantee a solid box, morphological operations are performed including imfill which cover any tiny gaps identified in the blue box area and bwareafilt that retains the most prominent linked region. Then, regionprops was applied to retrieve the bounding box coordinates as [w, h,x, y] which represented [width, height, top corner, left corner]. Second, the next stage aims to reduce and center the box size with 60%. This aims to reduce the presence of veins or tissues other than the lumen in the resulting reduced centered region, it in turn is an important process that paves the way for the next step to focus the scanning window search inside the carotid lumen. Third, a scanning window is defined that passes over the reduced centered region and calculates both mean intensity (µ) and standard deviation (**σ**) for each region it passes through as shown in Eq. (6) and Eq. (7). A scanning window of 30 pixels moves with a step of 5 to cover the central reduced region and obtain the best window for segmenting the lumen. If the mean intensity of the window is less than an intensity threshold of 60 and the standard deviation is less than a variance threshold of 15, the window is deemed a contender. The prospective window that minimizes the score that is the sum of its µ and **σ** is chosen as the best window among the others. The aim of the score measurement is to give priority to the dark and uniform areas, which are the essential key to accessing the carotid artery lumen. Finally, by finding the optimal window which has the minimum score (best Score), its dimensions are continually enlarged to better fit the entire carotid longitudinal lumen, which is usually broader than the tiny and uniform seed that was first discovered. The best window’s width is quadrupled, but its height stays the same. The expanded resulted window is horizontally re-centered according to the YOLOv11n blue box. To make sure the new box stays inside the image’s bounds, the coordinates are clipped. A last validation step is carried out on this expanded best window by checking it µ and **σ**. The segmentation based mask is defined by the expanded best window if this check is still satisfied. If not, the technique prevents expansion into inappropriate areas by reverting to the initial best window as the segmentation based mask.

6 $$\:\mu\:=\frac{1}{N}\sqrt{\sum\:_{i=1}^{N}\:{x}_{i}}$$

7 $$\:\sigma\:=\sqrt{\frac{1}{N}\sum\:_{i=1}^{N}\:\:{({x}_{i}-\:\mu\:)}^{2}}$$

### Carotid longitudinal lumen contouring

The lumen of carotid longitudinal section was segmented utilizing the active contour without edges^18,19^, which was accomplished using level set approaches, curve evolution, and the Mumford-Shah functional for segmentation. For tracking the interfaces, level set algorithms^20^ provide an immensely reliable and precise approach. The automated initialization of the Chan-Vese is an essential aspect to segment the carotid lumen. This guarantees that the Chan-Vese proceeds to develop from an area that has already been determined to be a very likely and recognizable section of the carotid lumen. The original image $\:{U}_{o}$is separated by the moving contour C into two homogeneous interior and exterior regions. The average intensity of pixels inside C is indicated by c_1_, and the average intensity outside C is indicated by c_2_ which are updated at every iteration as show in Eq. (9) and Eq. (10) where S is heaviside function and V is the level set function. The characteristics of the area in the image U (x, y) that the curve encircles modify the curve’s efflorescence. The following energy functional is introduced by the model, which aims to segment the image into sections based on pixel intensities. Chan-Vese algorithm attempt to reduce the energy functional $\:F\:\left({c}_{1},{c}_{2},C\right)$ as shown in Eq. (8) where µ ≥ 0, v ≥ 0, λ1, λ2 < 0, are fixed parameters.

8 $$\:F\:\left({c}_{1},{c}_{2},C\right)=\mu\:.Length\left(C\right)+\:{\lambda\:}_{1}{\int\:}_{inside\left(C\right)\:}\:\:{{[U}_{o}\:(x,\:y)\:-\:{c}_{1}\:]}^{2}dxdy\:+\:{\lambda\:}_{2}\:{\int\:}_{outside\left(C\right)\:}[{{U}_{o}\:(x,\:y)\:-\:{c}_{2}\:]}^{2}\:dxdy$$

9 $$\:{c}_{1}=\:\frac{\int\:{[U}_{o}\:(x,\:y)\:S\left(V\left(x,y\right)\right)dxdy}{\int\:\:S\left(V\left(x,y\right)\right)dxdy}$$

10 $$\:{c}_{2}=\:\frac{\int\:{[U}_{o}\:(x,\:y)\:(1-S\left(V\left(x,y\right)\right)\:)dxdy}{\int\:\:(1-S\left(V\left(x,y\right)\right)\:)dxdy}$$

### Testing environment

#### Machine tool

Different environments were used to cover this pipeline training, algorithms and computations. Kaggle environment with NVIDIA T4 GPU accelerated with CUDA and the Ultralytics framework 8.3.88 were used for localization of the carotid transverse and longitudinal lumens by fine-tuning YOLOv11n and applying cross validation utilizing high-performance computing. In contrast, the segmentation of the transverse and longitudinal carotid lumen (active contour and Chan-Vese ) were achieved using MATLAB (2019a) on Laptop Intel^®^ Celeron^®^ CPU 3955U and RAM of 8GB. This setup ensures that our suggested pipeline can be available in clinical settings with restricted computational resources. Jupyter notebook was also used in some intermediate stages of image processing and enhancement.

### Dataset and its preparation

Our proposed pipeline was implemented and validated using two datasets: one for longitudinal section and another for transverse section. The carotid transverse section dataset is publicly available on Mendeley Data^52^ contains 1100 US images of the common carotid artery transverse section with 709 × 749 × 3 dimensions, and 1100 expertly identified lumen masks. The carotid transverse images were captured using L13-3s linear probe on a Mindary UMT-500Plus US equipment. The carotid longitudinal section US images were freely accessible by Brno University’s SPLab^53^. A stringent 5-fold cross-validation procedure was developed to assess the model’s generalizability. We separated both transverse and longitudinal section images into three sets: training, validation, and a wholly independent hold-out test set. Specifically, 10% of the total images from both sections were separated before training and preserved untouched to serve as a final benchmark. The remaining 90% of the data underwent 5-fold cross-validation. The development pool was partitioned into five equal subsets. For each iteration, four subsets were used for training while the fifth acts as the validation set as shown in Table 1.This makes sure that every result provided on the test set shows how well the model works on data that it hasn’t seen before. This keeps the optimization and evaluation processes completely separate. The YOLOv11n model was trained on both carotid transverse and longitudinal with images size of 640 × 640 pixels. The training was applied with 100 epochs for transverse section and 300 epochs for longitudinal section, batch size of 16 and AdamW optimizer was selected with a weight decay of 0.0005 and dynamic learning rates which improves the generalization and stability of YOLOv11n training procedure. We built an early stopping strategy with a patience of 100 epochs, which allowed the training to be completed automatically after the validation loss stabilized. The localization model dynamically changed the momentum, which began at 0.9, and its starting learning rate, which had been configured at 0.01 to guarantee efficient convergence during the model training. Multiple data augmentation techniques were executed during YOLOv11n training to avoid overfitting and enhance robustness. Table 2 outlines the fixed hyperparameters utilized in YOLOv11n Model training in both transverse and longitudinal sections.

**Table 1:** Data partitioning for training, validation and independent testing.

Data Split	Percentage (%)	Transverse Images	Longitudinal Images
Development Pool	90%	990	76
Hold-out Test	10%	110	8

**Table 2:** Training hyperparameters for YOLOv11n model in transverse and longitudinal sections.

Hyperparameter	Value
Image size	640*640
Optimizer	AdamW
Initial Learning Rate	0.002
Momentum	0.9
Weight Decay	0.0005
Batch Size	16
Epochs	100 (Trans) / 300 (Long)
Patience	100
Augmentation	Mosaic, Albumentations, and Flip

A Sonix OP US scanner captured 84 B-mode US carotid longitudinal section images using linear array transducers with frequencies of 10 MHz and 14 MHz. The ground truth of the longitudinal section segmentation was determined by an expert radiologist specialized in carotid artery imaging. Both carotid transverse and longitudinal datasets contain the carotid binary lumen as the ground truth, but for YOLOv11n training, we need bounding box annotation to train the YOLOv11n network. So, a group of organized steps were applied to convert the carotid binary contour to appropriate YOLOv11n bounding box annotation. First, the binary contour points (X_b_, Y_b_) was deformalized to absolute pixel coordinates as X_b_^abs^ = X_b_ .W, Y_b_^abs^ = Y_b_ .H, where W, H are the width and height of the original image. Then the first bounding box four points ( Xmin, Ymin, Xmax, Ymax) was obtained by finding the minimum and maximum of X_b_^abs^ ,and Y_b_^abs^ as Eqs. (11), (12). Then a 10% padding of the width and height of the first bounding box (pad _x_, pad _y_) as Eqs. (13), (14) was, added to its both sides to make sure that carotid artery contour is totally inside the new padded bounding box which new coordinates are ( Xmin^P^, Ymin^P^, Xmax^P^, Ymax^P^) as shown in Eqs. (15), (16), (17), (18). Finally, these coordinates of the new padded bounding box were used to create the YOLOv11n text files [carotid class, Xc, Yc, width, height], the center of the bounding box Xc, Yc was computed as Eqs. (19), (20) and Width, Height was calculated as shown in Eqs. (21), (22).

11 $$\:Xmin,\:Ymin={min}({{X}_{b}}^{abs}\:\:,\:{{Y}_{b}}^{abs})$$

12 $$\:Xmax,Ymax=max({{X}_{b}}^{abs}\:\:,\:{{Y}_{b}}^{abs})$$

13 $$\:{Pad}_{x}=0.1\:(\:Xmax-Xmin)$$

14 $$\:{Pad}_{y}=0.1\:(\:Ymax-Ymin)$$

15 $$\:{Xmin}^{pad}={max}\left(\:0\:,\:Xmin-{Pad}_{x}\right)$$

16 $$\:{Ymin}^{pad}=max(\:0\:,\:Ymin-{Pad}_{y})$$

17 $$\:{Xmax}^{pad}={min}\left(\:W\:,\:Xmax+{Pad}_{x}\right)$$

18 $$\:{Ymax}^{pad}={min}\left(\:H\:,\:Xmax+{Pad}_{y}\right)$$

19 $$\:Xc=\:\frac{{Xmin}^{pad}+{Xmax}^{pad}}{2W}$$

20 $$\:Yc=\:\frac{{Ymin}^{pad}+{Ymax}^{pad}}{2H}$$

21 $$\:Width=\:\frac{{Xmax}^{pad}-{Xmin}^{pad}}{W}$$

22 $$\:Height=\:\frac{{ymax}^{pad}-{Ymin}^{pad}}{H}$$

## Results and discussion

### Evaluation metrics

#### Carotid transverse and longitudinal lumens localization

To evaluate and analyze the effectiveness of the YOLOv11n model used in detecting the carotid artery lumen and determining its bounding box in both the longitudinal and transverse sections, the following Standardized object detection measurements were used. These metrics represented in mean Average Precision (mAP) at various Intersection over Union (IoU) thresholds, precision and recall evaluations which offer an in-depth understanding of detection efficiency, accuracy, and reliability. According to Eq. (23), precision is the amount of precise positive forecasts amongst all of the model’s positive classifications where TP and FP are true positive and false positive. Thus, increasing its value indicates that the algorithm is more effective at recognizing real objects and less likely to improperly categorize background or undesirable samples as targets. Recall is the amount of real positive samples which is properly detects out of all ground-truth positive samples as FN is false negative, as explained in Eq. (24) where its increase enabling the framework to adequately identify all of the targets. Using an IoU of 0.5 serving as the minimal allowable overlap, the mAP at 0.5 IoU threshold (mAP 50) evaluates how closely the estimated bounding boxes match the ground truth as shown in Eq. (25) where N is the overall number of the dataset categories and AP_m_ is the average precision of class m. On the other hand, mAP50-95, as specified in Eq. (26), assesses the mean AP at a number of IoU thresholds, from 0.5 to 0.95 which measure the model performance at stricter overlap criteria where h is the IoU thresholds numbers and AP_m, t_ is the average precision for class m at a particular IoU threshold t.

23 $$\:Precision=\frac{TP}{TP+FP}$$

24 $$\:Recall=\frac{TP}{TP+FN}$$

25 $$\:{mAP\:}_{50}=\frac{1}{N}\sum\:_{m=1}^{N}{AP}_{50,m}$$

26 $$\:{mAP\:}_{50-95}=\frac{1}{N}\sum\:_{m=1}^{N}\frac{1}{h}\sum\:_{t=1}^{h}{AP}_{m,t}$$

#### Carotid transverse preprocessing framework

The preprocessing framework was evaluated using three image quality metrics to demonstrate that its enhancement process improved carotid transverse images details, increased contrast, and clarified the carotid lumen border edges. These metrics were measured on the original dataset images and also on the resulting images after going through all framework stages. First, the quantity of information or unpredictability in carotid transverse images was measured by entropy H^54^, as shown in Eq. (27) where the probability of a pixel with intensity level i occurring is denoted by p_i_. An image with a greater range of intensity levels, textures, and details will have a larger entropy which aids active contour approach in the decision-making process. Second, we compute the standard deviation (STD)^55^ of each pixel intensity value to enable quantifying global contrast as shown in Eq. (28), STD is **σ**, N is the total pixel’s numbers, x_i_ is the pixel intensity, and the image mean intensity µ. The higher value of standard deviation in carotid image indicates greater range of brightness levels and creation of large separation between dark and light areas which assist in reducing the image’s ambiguity for segmentation. Third, the image’s entire edge sharpness is measured by Mean Gradient Magnitude (MGM)^56^. MGM is evaluated by calculating the intensity changing rate at each pixel and then averaging these results. A higher mean gradient is generated by edges that are sharper and stiffer which supplying a precise track for the active contour to cover. The Sobel works to determine the horizontal G_x_ and vertical G_y_ derivatives and then used them to compute the average gradient magnitude G at all pixels as shown in Eq. (29).

27 $$\:H=\:-\sum\:_{i}{P}_{i}{{log}}_{2}{(P}_{i})$$

28 $$\:\sigma\:=\sqrt{\frac{1}{N}\sum\:_{i=1}^{N}\:\:{({x}_{i}-\:\mu\:)}^{2}}$$

29 $$\:G=\sqrt{{G}_{x}^{2}+\:{G}_{y}^{2}}$$

#### Carotid transverse and longitudinal lumens segmentation

Several performance segmentation metrics were applied to measure the efficiency and accuracy of the final phases outputs as active contour for transverse section and Chan-Vese for longitudinal section. These metrics were evaluated based on a comparison between the final contour C_F_ after the segmentation phases and the clinicians ground truth contours C_GT_. First, the dice index D_I_^57^ was measured as an important metric to evaluate the efficiency of the segmentation model by calculating the overlap between C_F_ and C_GT_ as shown in Eq. (30), so that reaching a percentage of 100% means that both contours match totally together. Second, sensitivity was evaluated by measuring the carotid artery lumen actual positive pixels that were correctly detected by the segmentation algorithm as shown in Eq. (31). Third, measuring the percentage correctly detected background pixels which knows as specificity as Eq. (32). Finally, calculating the accuracy as the total percentage of pixels that were successfully classified, both true actual lumen pixels and true background pixels as shown in Eq. (33).

30 $$\:{D}_{I}=\:\frac{2\:\left|\:{C}_{F}\cap\:{C}_{GT}\right|}{\left|\:{C}_{F}\right|+\:\left|\:{C}_{GT}\right|}$$

31 $$\:Sensitivity=\:\frac{\:\left|\:{C}_{F}\cap\:{C}_{GT}\right|}{\left|\:{C}_{GT}\right|}$$

32 $$\:Specificity=\:\frac{\:\left|\:Totalpixels\right|-\:\left|\:{C}_{F}\right|-\:\left|\:{C}_{GT}\right|+\left|\:{C}_{F}\cap\:{C}_{GT}\right|}{\left|\:Totalpixels\right|-\left|\:{C}_{GT}\right|}$$

33 $$\:Accuracy=\:\frac{\:\left|\:{C}_{F}\cap\:{C}_{GT}\right|+(\left|\:Totalpixels\right|-\:\left|\:{C}_{F}\right|-\:\left|\:{C}_{GT}\right|+\left|\:{C}_{F}\cap\:{C}_{GT}\right|)}{\left|\:Totalpixels\right|}$$

## Results

### YOLOv11n transverse and longitudinal lumen localization

**Fig. 6:**
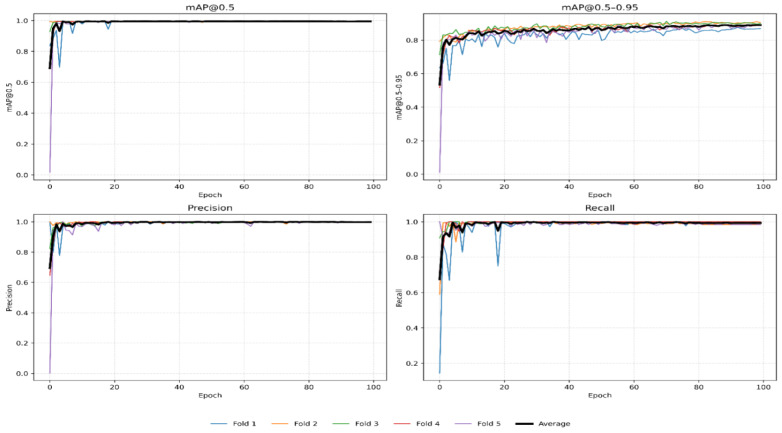
Transverse section Yolov11n training results for 5-fold cross validation.

**Fig. 7:**
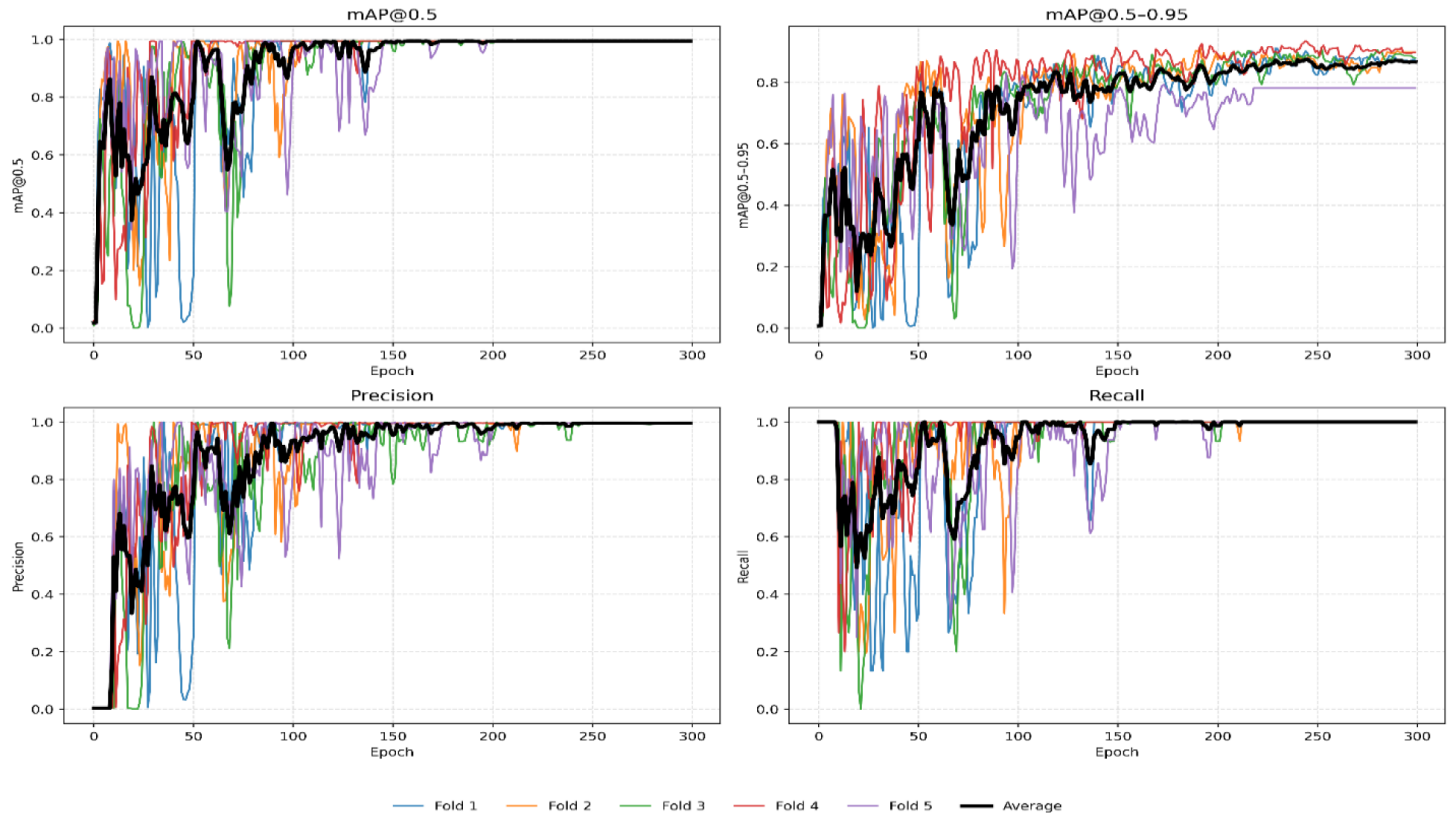
Longitudinal section Yolov11n training results for 5-fold cross validation.

Both carotid artery lumen sections including transverse and longitudinal were localized using YOLOv11n. The YOLOv11n model was fine-tuned on both transverse and longitudinal carotid artery datasets. The YOLOv11n localization model’s learning stability and convergence performance were assessed by measuring mAP@50, mAP@50–95, precision, and recall across the training period for both carotid sections. Figures 6 and 7 show these variations across the 5-fold cross-validation. The individual folds are depicted by colored lines, and the solid black line indicates the average performance across the 5 folds. In the transverse section, the model displayed very rapid and steady convergence, with all measures achieving a high-performance level within the first 40 epochs. This high level of consistency across the 5 folds shows that the carotid transverse lumen has particular features that YOLOv11n effectively captures. On the other hand, oscillations and fluctuations were more noticeable in the longitudinal section, especially in the first 150 epochs. This stochastic behavior is scientifically expected owing to the confined longitudinal dataset size and the intrinsic geometry complexity of the carotid longitudinal lumen. Furthermore, the efficiency of the early stopping mechanism is evident, while most folds used the full training epochs. Some folds (e.g., Fold 5, depicted in purple) reached ideal convergence substantially quicker than others. By stopping training once the validation loss became stable, the model successfully prevented overfitting, as demonstrated by this variation in convergence points. The final weights are well-generalized for invisible clinical data, as all folds eventually reached a steady and consistent level.

The YOLOv11n localization model’s exceptional robustness is demonstrated by its quantitative performance, which is represented in Table 3. For both carotid sections, a mean mAP@50 of 0.995 and an almost faultless recall of 1.0 were attained across the 5 folds. This confirms that the model is a reliable ROI extractor. Even with small bounding box variations, the whole carotid lumen will be safely contained within the ROI for the next segmentation stage. This is according to the deliberate use of a 10% padding during the localization ground truth preparation. Furthermore, the transverse section reached a high mAP@50–95 of 91.35%, although the longitudinal section showed a slight drop to 81.1%. This discrepancy is a direct result of longitudinal dataset size limits and the longitudinal section lumen’s complex and elongated architecture. However, for this hybrid pipeline, these accuracy levels are more than adequate to give a dependable and exact input for the Active Contour and Chan-Vese segmentation algorithms.

### Carotid transverse section preprocessing

Several metrics were calculated to evaluate the preprocessing stage for carotid transverse section images, including STD, entropy, and MGM. These metrics demonstrated the power of the preprocessing approach applied to the common carotid artery images in enhancing the images and making them more suitable for subsequent segmentation. As shown in the Table 4, the entropy was increased which indicates the information clarity and amount of data that increase on the carotid enhanced images that was obscure in the original carotid artery images. Furthermore, the increase in STD by approximately 60% in the enhanced images demonstrates the sharper contrast between the bright and dark parts of the carotid artery images, which in turn positively impacts the segmentation process. Finally, the nearly doubling of the MGM value indicates that the outer edges of the carotid artery lumen are clearly distinct, making it easier to track and identify by the active contour segmentation algorithm. This refinement stage is the key reason for the carotid transverse section’s high dice index. It creates a clear gradient map for the active contour model to proceed with.

**Table 3:** YOLOv11n transverse and longitudinal lumen localization results.

Section	Folds	mAP@0.5	mAP@0.5:0.95	Precision	Recall
Transverse section	Fold 1	99.5	91.35	99.94	1
	Fold 2	99.5	92.15	99.95	1
	Fold 3	99.5	91.08	99.98	1
	Fold 4	99.5	91.18	99.95	1
	Fold 5	99.5	90.97	99.95	1
	Mean ± Std	99.5	91.35 ± 0.47	99.95 ± 0.02	1
Longitudinal section	Fold 1	99.5	84.68	99.37	1
	Fold 2	99.5	81.44	99.34	1
	Fold 3	99.5	77.37	99.37	1
	Fold 4	99.5	81.64	99.37	1
	Fold 5	99.5	80.35	99.34	1
	Mean ± Std	99.5	81.1 ± 2.63	99.36 ± 0.02	1

**Table 4:** comparison of quality metrics between carotid artery original images and our enhanced images.

Metric	Carotid transverse original images	Carotid transverse enhanced images
Entropy	7.21	7.83
STD	42.42	68.58
MGM	0.02	0.05

**Fig. 8:**
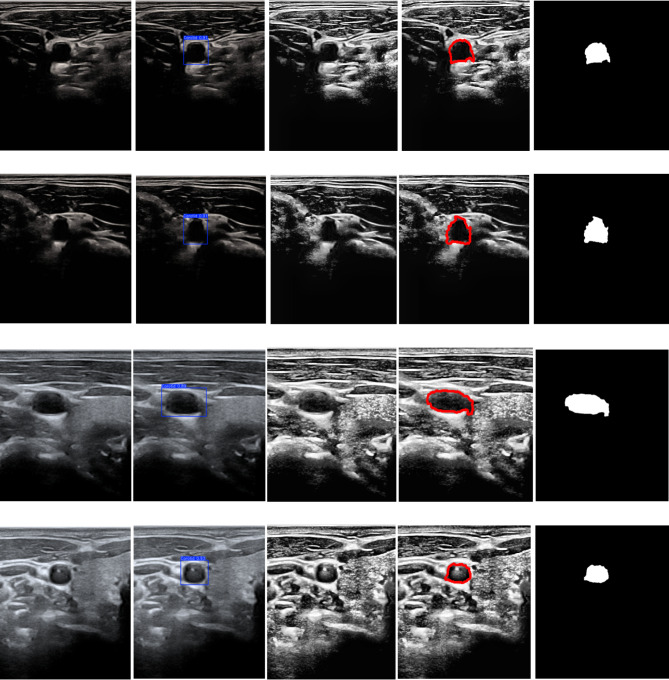
Carotid transverse lumen segmentation. The first column provides the original images. The second column provides the carotid transverse lumen localization using YOLOv11n showed as a blue bounding box with the percentage value of the detection confidence score. The third column provides the enhanced carotid transverse images. The fourth column provides the red contoured carotid transverse lumen using active contour approach. The fifth column provides the carotid binary segmented transverse lumen.

The results of the localization, preprocessing, and segmentation of the carotid transverse section are shown in Fig. 8. The first column contains the original images, while the second column contains the YOLOv11n bounding box that defines the carotid transverse lumen and later used as a preliminary box to facilitate and increase the efficiency of the lumen segmentation, as well as to reduce complex calculations that would be performed on unrelated areas hindering the segmentation process. The third column contains the enhanced carotid transverse images which improves the lumen area and highlights its boundaries. Therefore, the final segmentation process is carried out on the enhanced images with the preliminary blue box using the active contour. The results of image enhancement and the bounding box initialization are demonstrated when comparing the generated contour with the ground truth, achieving a great dice index of 90.8% and superior sensitivity, specificity, and accuracy of 99.2%, 99.8%, and 99.6%.

**Fig. 9:**
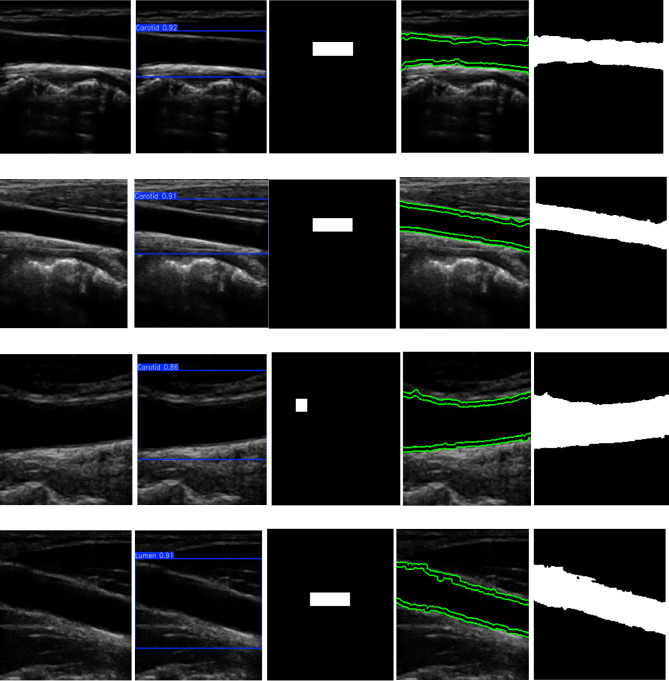
Carotid longitudinal lumen segmentation. The first column provides the original images. The second column provides the carotid longitudinal lumen localization using YOLOv11n showed as a blue bounding box with the percentage value of the detection confidence score. The third column provides the second white bounding box generation which acts as the initial mask for the segmentation procedure. The fourth column provides the green contoured carotid transverse lumen using active contour approach. The fifth column provides the carotid binary segmented longitudinal lumen.

The second column in Fig. 9 shows the results of the carotid longitudinal lumen localization using YOLOv11n, which was not qualified as a preliminary contour for the longitudinal lumen segmentation. Therefore, the third column in Fig. 9 shows the white bounding box that was derived based on the blue YOLOv11n box, which is in the best opaque and homogeneous area within the carotid longitudinal lumen, making it the best preliminary box for the segmentation process using the Chan-Vese. In the third column, the (first, second and fourth) rows, the boxes are extended and centered, as they satisfy the µ, and check. As for the third column, third row, this contains a white bounding box that didn’t meet the criteria. Figure 9, column 4, shows the results of the Chan-Vese algorithm segmentation, which shows a precise delineation of the carotid longitudinal lumen borders with a solid green contour, without containing any other areas outside the lumen. The fifth column in the same figure contains the carotid longitudinal binary segmented lumen. This precise borders delineation of the carotid longitudinal lumen is also consistent with the quantitative assessments, achieving a dice index of 94.9%. This indicates the percentage of overlap of the green contour resulting from the Chan-Vese with the ground truth contours. The high sensitivity of 90.6% shows that the algorithm was able to correctly infer most of the carotid longitudinal lumen pixels and the excellent specificity of 99.9% also indicates that it was able to pass and identify the background pixels surrounding the carotid longitudinal lumen superiorly and avoiding over segmentation. The segmentation algorithm also achieved an overall accuracy of 97.7%, which indicates the overall efficiency of the system.

### Ablation study

**Fig. 10:**
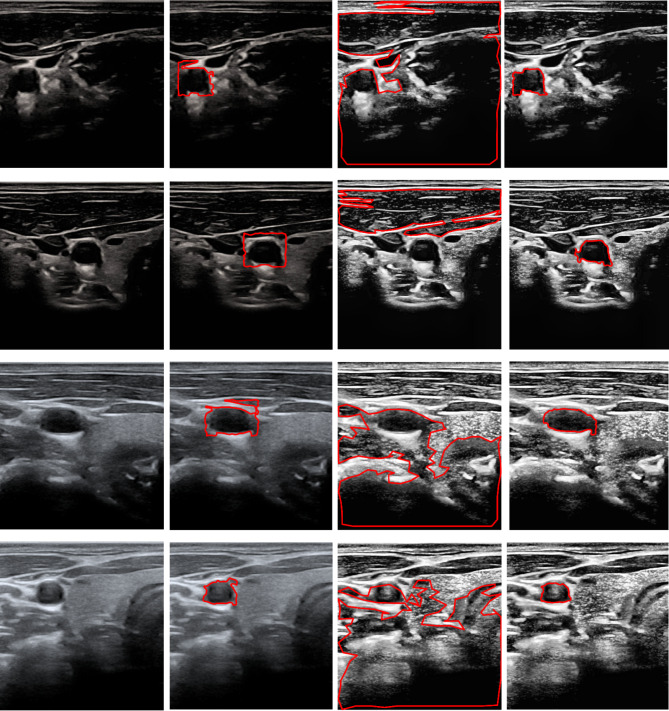
Carotid transverse section ablation study. The first column provides the original images. The second column provides the final output of ablation 1 that applied the YOLOv11n blue bounding box on the original image without preprocessing procedures. The third column provides ablation 2 that shows the failure of global segmentation on the enhanced image without YOLOv11n. The fourth column confirms the precision of our full proposed pipeline.

**Fig. 11:**
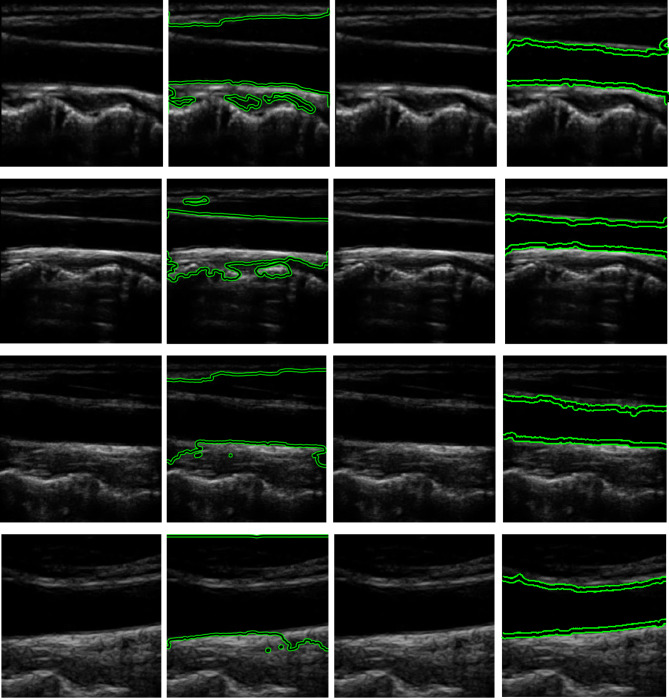
Carotid longitudinal section ablation study. The first column provides the original images. The second column provides the final output of ablation 1 that applied the chan vese segmentation using the initial YOLOv11n blue bounding box. The third column provides ablation 2 that shows the complete convergence failure of full-frame segmentation. The fourth column confirms the precision of our full proposed pipeline.

The proposed hybrid pipeline’s architectural integrity was extensively assessed using multi-stage ablation research to measure the distinctive contributions of each pipeline stage. This empirical analysis, described numerically in Table 5, demonstrates the importance of combining deep learning-based detection with enhanced contour-based segmentation. By isolating the effects of YOLOv11n ROI identification, automated padding mask, and preprocessing filters, the study presents a clear picture of how each module inhibits US-related artifacts. The qualitative results of these experiments are clearly depicted in Fig. 10 for the carotid transverse section and Fig. 11 for the longitudinal section. This demonstrates a strong relationship between algorithmic improvements and segmentation accuracy. The carotid transverse section findings from the analysis demonstrate the need for localized limitations and regional filtering in handling high levels of speckle noise. As shown in Fig. 10, the entire suggested pipeline had an ideal dice index of 90.76%, indicating a high level of anatomical authenticity. However, after the preprocessing stage was removed in Ablation 1, the dice index dropped significantly to 84.82%. Visually, this setup was highly distracted by intraluminal aberrations and blood flow echoes, causing the active contour to diverge from the actual carotid artery lumen. The most persuasive proof for the pipeline’s architecture emerged in Ablation 2, where excluding the YOLOv11n-based ROI and applying the active contour on the whole enhanced images resulted in a total accuracy collapse of 5.31%. Applying the active contour on the whole image made the algorithm unable to distinguish the carotid lumen from nearby structures such as the jugular vein and neck muscles, resulting in the expanded and clinically incorrect contours shown in the third column of Fig. 10.

**Table 5:** Ablation study performance metrics. The (–) symbol denotes the reference proposed system, and Failed indicates instances where the algorithm reached numerical instability.

Section	Configuration	Dice Index (%)	Sensitivity (%)	Specificity (%)	Accuracy (%)	*p*-value (vs. Proposed)
Transverse	Ablation 1 (No Preprocess)	84.82	89.26	99.4	99.18	< 0.01
	Ablation 2 (No YOLO ROI)	5.31	23.46	89.61	24.91	< 0.0001
	Proposed	90.76	86.21	99.8	99.54	–
Longitudinal	Ablation 1 (No Padded Mask)	71.17	97.98	73.04	79.82	< 0.001
	Ablation 2 (No YOLO ROI)	Failed	Failed	Failed	Failed	< 0.0001
	Proposed	94.9	90.57	99.9	97.67	–

The carotid longitudinal section study demonstrates the superiority of the suggested automated mask generation over traditional bounding box procedures. The longitudinal proposed hybrid pipeline achieved a superior dice index of 94.90%. Ablation 1 was applied by using the YOLOv11n blue bounding box without special padding, which resulted in a significant performance loss to 71.17%. As illustrated in the second column of Fig.11, this setup suffered from boundary adherence at the artery’s distal boundaries. The hard rectangular initialization does not readily conform to the stretched lumen shape. More crucially, Ablation 2’s global initialization (using a full-image mask) did not achieve complete numerical convergence. The Chan-Vese energy function failed to extract the carotid boundaries due to the strong background noise, resulting in the non-finite findings labeled “Failed” in Table 5. This failure demonstrates that the YOLOv11n-driven ROI is essential for maintaining the mathematical stability of level-set evolution in the carotid longitudinal section. The p-values in Table 4 demonstrate the proposed system’s superiority at a high level of significance (*p* < 0.0001). Ablation 1 had occasionally higher sensitivity than the proposed system. However, a critical analysis indicates that this was not a sign of improved function but rather the result of over-segmentation and leakage outside the carotid borders. Because the contour in Ablation 1 frequently spreads uncontrollably into the surrounding tissue. It naturally encompassed the whole true-positive area, inflating the sensitivity measure while also lowering the overall dice index and specificity. These findings show that the combination of YOLOv11n’s spatial intelligence and the improved contouring algorithms makes a strong barrier against the natural limits of US imaging. This makes sure that the final segmentation is both clinically precise and mathematically stable. Comparative analysis of various studies for carotid artery segmentation in transverse section and longitudinal section are shown in Tables 6 and 7.

**Table 6:** Comparative analysis of various studies for carotid artery segmentation in transverse mode.

Method	Year	Method	# images	Dice coefficient (%)	Type
Yang et al.^17^	2012	GVF-Snake	110	92.7	Semi-automated
Tang et al.^18^	2013	Level Set	56	90.2	Semi-automated
Ukwatta et al.^19^	2013	Modified sparse field level set	21	90.6	Semi-automated
Andres et al.^20^	2013	Surface graph cuts	12	84 & 66.7	Semi-automated
Md. Murad et al.^21^	2014	Distance regularized level set	5 subjects	89.82	Semi-automated
Md. Murad Hossain et al.^22^	2015	Distance regularized level set + Ellipse Fitting	10 subjects	88.78	Semi-automated
Narayan et al.^23^	2015	Hypoechoic region analysis + Hessian blob	41	87.4	Automated
Arias-Lorza et al.^24^	2015	Coupled Optimal Surface Graph Cuts	57	89	Semi-automated
Hamidreza et al.^25^	2015	Level set	14	85	Semi-automated
Danilo et al.^26^	2016	k-means + Active contour	362	78 & 61	Automated
Luo et al.^27^	2019	level set + Double adaptive threshold	283	88	Semi-automated
Zhou et al.^31^	2019	U-Net	144	92.8	Semi-automated
Xie et al.^33^	2019	Single-path & Multi-path U-Net	302	NS	Automated
Jiang et al.^32^	2021	2 channel U-Net + Adaptive Triple Dice Loss	224	89	Semi-automated
Zhu et al.^28^	2022	Improved Optimal Surface Graph Cuts	NS	89.6	Semi-automated
Jonnala et al.^35^	2023	Improved U-Net	2165	87.03	Automated
Lin et al.^34^	2023	CSWin Transformer	NS	90.8	Automated
Chen et al.^58^	2024	Unsupervised Shape & Texture-based GAN) + Pre-trained U-Net	224	85.7	Automated
Liu et al.^29^	2025	CANet (Modified U-Net (	512 cases	92.41	Automated
Yan et al.^30^	2025	YOLOv7 +SAM	400 cases	83.7	Automated
**Proposed method**	**2026**	**YOLOv11n + Active contour**	**1100**	**90.76**	**Fully automated**

**Table 7:** Comparative analysis of various algorithms for carotid segmentation in longitudinal Section.

Method	Year	Method	Modality	# images	Dice coefficient (%)	Type
Md. Murad Hossain et al.^37^	2013	Distance-Regularized Level Set	3D US	6 patients	86.4	Semi-automated
Loizou et al.^59^	2015	2D Snakes	2D US	300	NS	Semi-automated
Kumar et al.^38^	2019	Active Oblongs	2D US	84	93.35	automated
Biswas et al.^43^	2019	Encoder–Decoder CNN + Fully Convolutional Network	2D US	407	93	automated
Xie et al.^60^	2020	U.Net	2D US	2156	94.3	automated
Gagan et al.^39^	2022	Basis splines based active contour	2D US	84	91.7	automated
Kiernan et al.^41^	2024	Mask R-CNN	2D US	101 patients	85.7	automated
**Proposed method**	**2026**	**YOLOv11n + Chan-Vese**	2D US	**84**	**94.9**	**Fully automated**

## Discussion

Segmentation of the carotid lumen in US images is the initial step in diagnosing prospective cardiovascular disorders such as atherosclerosis. However, automatic carotid lumen segmentation remains a difficulty due to poor image quality and the existence of additional factors in the carotid images like stenosis, the jugular vein, and some abnormalities, which impair the accuracy of the results. This article introduced a hybrid pipeline for fully automated segmentation of the carotid transverse and longitudinal lumen without necessitating user interaction. The automated YOLOv11n detection phase had an inference time of 11 milliseconds (conducted on a T4 GPU). Crucially, the segmentation stage, which was carried out on an Intel^®^ Celeron^®^ CPU, took only 0.8 s for the transverse section. Although the longitudinal Chan-Vese evolution took 23.6 s. It is still quite efficient for clinical situations involving limited equipment resources, where manual segmentation might require much longer. This proves that the suggested pipeline is not only accurate but also feasible for real-world use on small medical workstations. However, several studies have used semi-automated algorithms to segment the carotid artery, which is a major obstacle that consume time and makes them susceptible to subjective errors^17,18,28,27,61^. Accurate location settings of the carotid automated initial contour used is very important for determining the efficiency, speed and accuracy of the subsequent segmentation stage. Many studies have encountered difficulties and errors due to the inaccuracy of the initial mask used for the segmentation process^15,41,25^. Our study’s findings highlight the medical prospects of the hybrid pipeline, which combines the mathematical rigor of contour models with the spatial intelligence of deep learning. The ultimate validation of the structure came from applying the ablation study. The segmentation models failed when the YOLOv11n-ROI or any intermediate refinement phases were removed. It demonstrates that the success of our pipeline is the consequence of a carefully designed synergy. Each phase of our hybrid pipeline, from preprocessing to padded mask generation, acts as a necessary prerequisite for numerical stability and contour leakage elimination.

Comparative analysis of previous studies in Table 6 reflects the evolution of carotid transverse lumen segmentation methods from traditional semi-automated methods^17,18^ to end-to-end deep learning methods^35,58^. Zhou et al. ^31^ achieved a high dice coefficient in segmenting the carotid transverse lumen, but it still required human intervention in determining its initial segmentation contour. Furthermore, their slicing from 3D images to 2D images, due to the difficulty of training 3D networks, resulted in a loss of spatial coherence. The use of Dynamic CNN + UNet means their system is not fully classified as an end-to-end deep learning algorithm. Advanced models like CANet and CSWin Transformer have achieved a high dice index, but they require high computational resources, limiting their applicability in limited medical devices. However, the YOLOv7 + SAM method^30^ is considered as an automated framework, but their dice coefficient drops to 83.7%. It indicates that direct reliance on SAM without precise local boundary optimization may not be sufficient for accurate segmentation. Our transverse pipeline is distinguished by the lightweight YOLOv11n used in the localization phase, followed by Geometry-based refinement active contour, resulting a dice index of 90.76%. This pipeline achieves a complete integration of fully automation, precision, and architectural simplicity compared to other studies.

Table 7 shows the limited number of studies that discussed carotid longitudinal lumen segmentation compared to transverse section segmentation studies. This is attributed to the scarcity of online longitudinal databases and the reliance of most current research on proprietary databases. Early methods relied on semi-automated longitudinal lumen segmentation applied to low patient volumes^37,59^. With the advent of deep learning, Biswas et al.^43^ presented an Encoder–Decoder CNN model on 407 images, but it focused solely on segmentation without comprehensive end-to-end integration. Xie et al. ^60^ also used U-Net on a relatively large dataset (2156 images), but it required data preprocessing and did not clearly address the issue of longitudinal variance. More recent models, such as Mask R-CNN^41^, have improved automation, but they require significant computational resources, and the data is not publicly available. Our longitudinal pipeline presents an automated and precise segmentation method with a dice index of 94.9%.

Our proposed hybrid pipeline was implemented on specific datasets for both transverse and longitudinal sections. Cross-validation demonstrated that the proposed pipeline exhibits a high degree of stability within the dataset used. Therefore, expanding the datasets used to include multicenter data and different imaging devices will enhance the model’s robustness and support its adoption on a broader clinical scale. The limited number of longitudinal section studies also provides a direct incentive to expand future comparisons as additional data and methods become available in this area. Furthermore, the proposed framework will be applied to segment the carotid plaque in both sections. This will provide a more comprehensive vascular characterization, supporting clinical assessment decisions. Our proposed framework is designed to be practical and clinically integration. It relies on a clear separation between the spatial identification and fine-tuning phases. This design enables future expansion of the system to include additional analytical modules without necessitating a complete overhaul of the basic structure. Our pipeline showed impressive performance on a standard Intel^®^ Celeron^®^ CPU. Despite that, modern deep learning frameworks usually require high end GPU clusters for inference. The ability to execute near real time transverse segmentation (< 1 s) on such limited hardware is a crucial finding for point of care US devices. The high precision attained in the carotid artery’s lengthened geometry justifies the longer execution time (23.6 s) necessary for the longitudinal Chan-Vese evolution. This automated method provides a standardized, repeatable tool that can be implemented on current hospital workstations without needing costly hardware changes.

## Conclusion

Automated CCA segmentation is crucial when evaluating the presence of atherosclerosis, which can lead to stroke. We have proposed a fully automated segmentation pipeline for both the carotid transverse and longitudinal lumens. The carotid lumen localization of both sections is achieved using YOLOv11n. The active contour is applied to segment the carotid transverse lumen, initialized with the resulting YOLOv11n bounding box after applying a group of enhancement stages to the carotid transverse US images. The enhancement stages are critical for enhancing the carotid lumen edges contrast and reducing the speckle noise, which presents an optimal energy landscape for active contour delineation. The Chan-Vese is applied to segment the carotid longitudinal lumen, initialized with a second generated bounding box from the YOLOv11n longitudinal blue bounding box. The second generating bounding box helps the Chan-Vese to only segment the carotid artery lumen and avoid segmenting the jugular vein, unlike other state-of-the-art methods. The proposed segmentation pipeline achieved an accuracy of 97.7% for the longitudinal section and 99.6% for the transverse section. These results indicate the great ability of the pipeline to precisely segment the CCA lumen, outperforming numerous existing methods.

## Data Availability

The dataset in the manuscript is from public datasets. The data were sourced from two distinct datasets:1. Carotid Transverse Section Dataset: This dataset is publicly available via the Mendeley Data repository (DOI: 10.17632/d4xt63mgjm. (1) and can be accessed directly through the link: https://data.mendeley.com/datasets/d4xt63mgjm/. 2. Carotid Longitudinal Dataset: This dataset is publicly available via the Zenodo Data repository (DOI: 10.5281/zenodo.17552717) and can be accessed directly through the link: 10.5281/zenodo.17552717.
